# Hepatitis B prevalence among blood donors at Edward Francis Small Teaching Hospital, Gambia

**DOI:** 10.11604/pamj.2023.44.66.38656

**Published:** 2023-02-02

**Authors:** Ramatoulie Darboe, Sheikh Omar Bittaye, Saydiba Tamba, Ramou Njie

**Affiliations:** 1 Internal Medicine Department, School of Medicine and Allied Health Sciences, University of The Gambia, Serekunda, The Gambia,; 2 Internal Medicine Department, Edward Francis Small Teaching Hospital, Banjul, The Gambia

**Keywords:** Hepatitis B, blood donors, prevalence, replacement, voluntary, Gambia

## To the editors of the Pan African Medical Journal

Hepatitis B is a significant health problem in sub-Saharan Africa. Worldwide, an estimated more than 2 billion people are infected with Hepatitis B Virus (HBV) with 350 million (5%) chronic carriers [1,2]. HBV infection is transmitted via blood, contact with body fluid and mother-to-child and is highly infective. Among these different transmission routes, transmission through blood transfusion is the most deliberate way of transmission which can be prevented. Despite the high prevalence of hepatitis B in The Gambia, there is limited research regarding its prevalence among the replacement and Voluntary Non-Remunerated Blood Donors (VNRD). This study evaluates the prevalence of hepatitis B virus among blood donors at Edward Francis Small Teaching Hospital (EFSTH).

This was a descriptive, cross-sectional study conducted at EFSTH in The Gambia, from 20^th^ February to 30^th^ April 2021. The study recruited 449 blood donors, mostly males 447 (99.6%) between the 21-40 year age group 362 (80.6) (Table 1). This finding is similar to an earlier study done in The Gambia in 2013 [3]. Other studies within the sub-region [2,4] also confirmed similar findings. The reasons for this could be due to some socio-cultural factors and beliefs that may play an important role (2).

**Table 1:** characteristics of blood donors in Edward Francis Small Teaching Hospital

Variable	n=449
Age (years) mean	31.8
Median	31 (17-57)
**Age groups**	
16-20	26 (5.8)
21-30	192 (42.8)
31-40	170 (37.9)
41-50	46 (10.2)
>50	12 (2.7)
Missing	3 (0.7)
**Vaccination status**	
Likely vaccinated	230 (51.2)
Unlikely vaccinated	217 (48.3)
Missing	2 (0.4)
**Sex**	
Male	447 (99.6)
Female	2 (0.5)
**Ethnicity**	
Mandinka	138 (30.7)
Fula	64 (14.3)
Wollof	81 (18.0)
Jola	90 (20)
Others	76 (16.9)
**Level of education**	
Tertiary	56 (12.5)
High school	151 (33.6)
Middle school	91 (20.3)
Primary school	26 (5.8)
Arabic	65 (14.5)
None	58 (12.9)
Missing	2 (0.4)
**Occupation**	
Civil Servant	111 (24.7)
Student	32 (7.1)
Business	90 (20)
Labourer	66 (14.7)
Farmer	12 (2.7)
Others	123 (27.4)
None	15 (3.3)
**Number of donations**	
New	206 (45.9)
Regular	242 (54)
Missing	1 (0.2)
**Type of donation**	
Replacement	363 (80.8)
Family	246 (67.8)
1^st^ degree relative	144 (58.5)
2^nd^ degree relative	51 (20.7)
3^rd^ degree relative	51 (20.7)
Non-family	117 (32.2)
Voluntary	86 (19.2)
**Blood group**	
O positive	289 (64.5)
A positive	72 (16.1)
B positive	65 (14.5)
AB positive	10 (2.2)
A negative	2 (0.5)
B negative	2 (0.5)
O negative	7 (1.6)
AB negative	1 (0.2)
**Hepatitis B status**	
Negative	404 (90)
Positive	45 (10)

As a result of the shortage of volunteer donors, family blood donors are commonly used to donate blood to their relatives admitted in hospitals. In 2002, greater than 60% of blood donors came from replacement/family blood donors in Africa as per World Health Organization (WHO) estimates [4]. This study showed similar findings as most of the blood donors were family/replacement blood donors 246 (67.8%) and mostly of first degree relatives. Other studies had similar findings [4,5]. However, in studies done in Kenya [4] and Pakistan [6], voluntary blood donors were more common. The prevalence of hepatitis B among replacement blood donors (11%) was higher compared to VNRD (5.9%) in this study. This is consistent with another study done in Tanzania [7]. This confirms the low numbers of willing healthy voluntary blood donors who are at lower potential risk of transfusion transmissible infection and thus families resort to family/replacement donors only in emergency situation when there is fear of death of their relatives.

The prevalence of hepatitis B among the blood donors in this study was 10%. This is lower than a previous study done in 2013 (13%) [3]. The reasons for this could be due to: 1) the increasing age of the hepatitis B vaccinated cohort now at ≤32 years as compared to ≤23 years in 2013; 2) most of the patients in this study were also within the vaccinated age group 230 (51.2) and; 3) prevalence of hepatitis B was also lowest among blood donors with 30 years or less (6.9%). The overall prevalence in this study was similar to that of Sierra Leone [5] and Ghana [8]. The prevalence of hepatitis B also increased with age and was highest (25%) in blood donors above 50 years of age. This could be explained by the aging of the non-vaccinated age groups as nationwide hepatitis B vaccination was started in 1990 in The Gambia and coverage has now exceeded 90% [9]. The prevalence of hepatitis B amongst new blood donors (17%) was also significantly higher compared to regular blood donors (7%) in this study. This confirms the fact that regular blood donors most likely may have been to health facilities and got screen for hepatitis B before as compared to first time blood donors who most likely have never been screened for hepatitis B. This finding is similar to a study done in Sierra Leone [5].

Blood donors who tested positive for hepatitis B were older 34.6 vs 31.5 years (0.009) and less likely to be part of the hepatitis B vaccinated cohort (p=0.010) as compared to patients with negative hepatitis B. This confirms the fact that most of the blood donors who tested positive for hepatitis B are part of the non-vaccinated hepatitis B cohort and thus may not have been vaccinated. This situation is different from that observed in some countries within the sub-region. In Sierra Leone and Ghana, HBV infection among the blood donors was associated with younger age [5,8]. The reason for this could be that Sierra Leone and Ghana started nationwide hepatitis B vaccination in 2007, 2001 respectively as compared to 1990 for The Gambia [10].

## Conclusion

The study showed a decreasing prevalence of hepatitis B among the blood donors in EFSTH which could be explained by the increasing age of the vaccinated age group who are the majority blood donors. It also confirms the urgent need for aggressive mobilization and recruitment of healthy, young, regular and voluntary non-remunerated blood donors who are at lower potential risk of HBV infection.
